# A new anatomical finding: the paramastoid diverticulum of the sigmoid sinus

**DOI:** 10.1007/s00276-024-03558-9

**Published:** 2024-12-30

**Authors:** Mugurel Constantin Rusu, Corneliu Toader, Răzvan Costin Tudose

**Affiliations:** 1 Division of Anatomy, Faculty of Dentistry, “Carol Davila” University of Medicine and Pharmacy, Bucharest, 020021 Romania; 2 Division of Neurosurgery, Department 6—Clinical Neurosciences, Faculty of Medicine, “Carol Davila” University of Medicine and Pharmacy, Bucharest, RO-020021 Romania; 3 Clinic of Neurosurgery, “Dr. Bagdasar-Arseni” Emergency Clinical Hospital, Bucharest, RO-041915 Romania

**Keywords:** Dural sinuses, Jugular process, Paramastoid process, Suboccipital region, Posterior cranial fossa

## Abstract

**Purpose:**

The sigmoid sinus (SS) is a major surgical landmark. The paramastoid process (PMP) occurs rarely. Inferior diverticula of the SS were not found or reported previously. We aimed to determine the incidence and detailed anatomy of such morphology of the SS.

**Methods:**

Archived angioCT files of 25 males and 25 females were used. The morphology of the SS was checked on planar sections and by three-dimensional volume renderings.

**Results:**

In 3 female cases (6%), inferior paramastoid diverticula of the SS (PMDSSs) were found, two on the right and one on the left. They were all protruding on the inferior side of the jugular process of the occipital bone. Their heights and inner diameters were, respectively, 9.94/11.01 mm, 8.21/4.85 mm and 5.97/8.72 mm. A high jugular bulb was also found on that side in each case. Each PMDSS had a thin or dehiscent bottom. They were closely related to condylar veins, the occipital artery, the vertebral artery and its venous plexus.

**Conclusion:**

The PMDSS should not be mistaken as a PMP to avoid surgical lesions of the SS. The PMDSS is an unexpected landmark in the suboccipital region.

## Introduction

Meckel’s paramastoid process (PMP) is also termed the paraoccipital, paracondylar, parajugular, juxtamastoid, jugular, paramastoid and estiloid process, as documented previously [8]. It is located on the inferior side of the jugular process of the occipital bone, and it rarely occurs (0.5-2%) [8]. The PMP is lateral to the occipital condyle, medial to the mastoid process and posterior to the jugular fossa of the petrous bone [8]. It may articulate with the transverse process of the atlas [6]. The PMP belongs to a group of congenital craniovertebral junction anomalies [4].

Detailed knowledge of the possible anatomic variations of the craniocervical junction is essential for neck and cranial surgery [8]. Therefore, preoperative identification of a PMP is critical [8]. Several structures were listed by Schumacher et al. (2018) to be spared when a PMP is removed: the vertebral artery, facial nerve, and contents of the jugular foramen [8].

The sigmoid sinus (SS) courses on the upper side of the jugular process of the occipital bone in a groove to reach the jugular foramen. The SS is a significant landmark in different surgical approaches, either of the cranial fossae and nerves or the ear structures within the petrous bone [5, 10]. The diverticula of the SS are described as intruding into the mastoid. To our best knowledge, an inferior or paramastoid diverticulum of the SS has not been reported previously. We serendipitously found such a diverticulum, and we further designed and performed a brief study of its prevalence and detailed anatomy.

## Materials and methods

A sublot from a previous study [3] was used. It consisted of 50 archived angioCT files from 25 males and 25 females. The CT examination was performed on a 64-slice CT Somatom Definition As (Siemens), with a rotation time of 0.5 s, using a pitch of 1.2 and collimation of 1.2 mm. The technical parameters were detailed previously [3]. Anatomical variants were documented with Horos v3.3.6 software for macOS (Horos Project, Annapolis, MD, USA), on planar or curved planar sections, and via three-dimensional volumetric renderings. The principles of the Declaration of Helsinki were used to conduct the research. The Ethics Committee (affiliation #3) approved the study (approval no. 2093/1 March 2022). The presence and the anatomical details of inferior diverticula of the sigmoid sinuses were recorded.

## Results

**Fig. 1:**
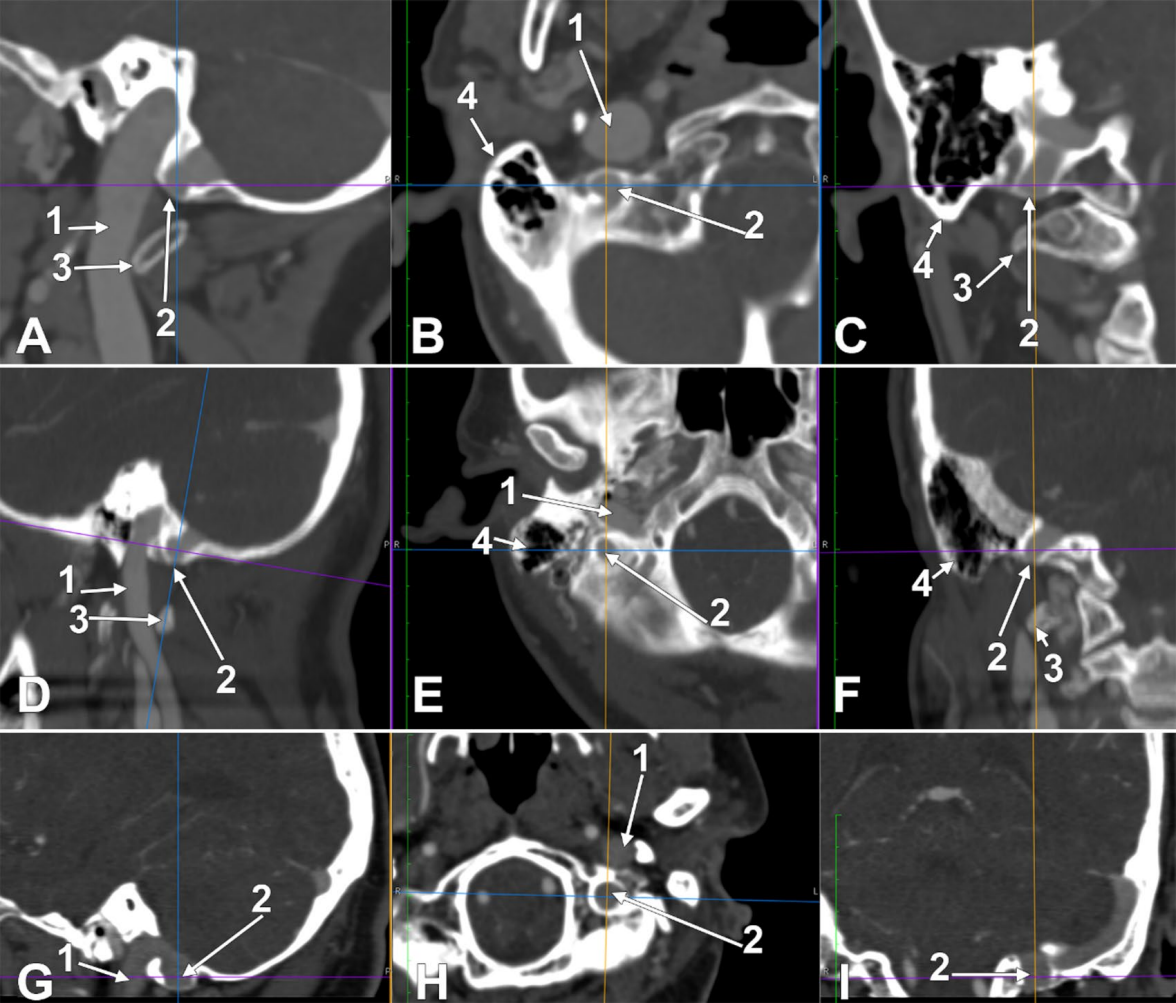
Correlated two-dimensional slices in the anatomical planes, sagittal (**A**, **D**, **G**), axial (**B**, **E**, **H**) and coronal (**C**, **F**, **I**) in the three cases with a paramastoid inferior diverticulum of the sigmoid sinus. The axial sections are viewed inferiorly, and the coronal sections are viewed anteriorly. The anatomical variation is on the right side in cases #1 (**A**, **B**, **C**) and #2 (**D**, **E**, **F**). The variant in case #3 (**G**, **H**, **I**) is on the left side. (1) internal jugular vein; (2) paramastoid diverticulum of the sigmoid sinus; (3) transverse process of the atlas; (4) mastoid process

**Fig. 2:**
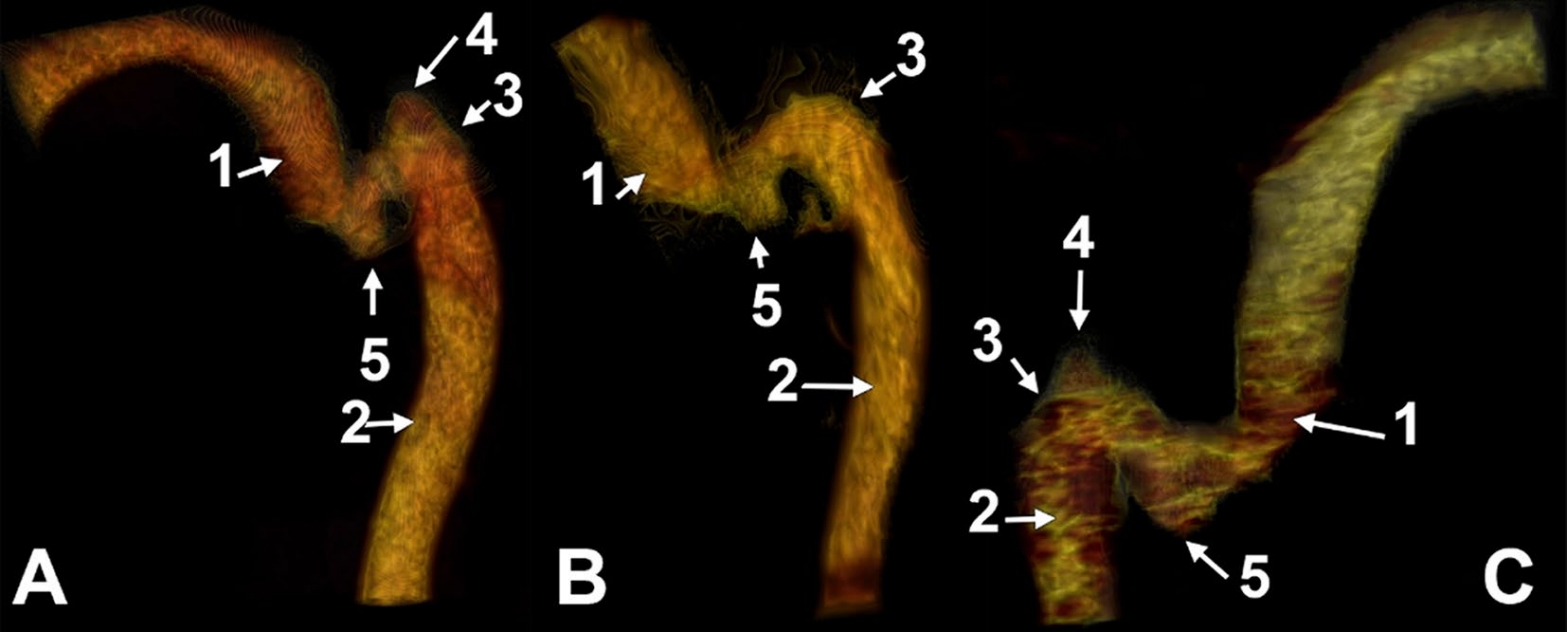
Three-dimensional volume renderings of the inferior paramastoid diverticula of the sigmoid sinuses in cases #1 (A, right side, lateral view), #2 (B, right side, lateral view) and #3 (C, left side, lateral view). (1) sigmoid sinus; (2) internal jugular vein; (3) jugular bulb; (4) superior diverticulum of the jugular bulb; (5) inferior paramastoid diverticulum of the sigmoid sinus

In three female cases (Figs. 1 and 2) there was found a peculiar anatomic variation of the sigmoid sulcus and sinus: an inferior recess of the sulcus was filled by an inferior diverticulum of the sigmoid sinus and projected inferiorly from the jugular process of the occipital bone. This leads to a 6% general and 12% prevalence in females.

Due to this topography, that diverticulum was termed „paramastoid diverticulum of the sigmoid sinus” (PMDSS). The variant was unilateral in all the cases. In cases #1, #2 and #3, the heights (measured from the bottom of the sigmoid sulcus) and inner diameters of the PMDSSs were, respectively, 9.94/11.01 mm, 8.21/4.85 mm and 5.97/8.72 mm. In each of these cases, a high jugular bulb was also found on the side with PMDSS (Fig. 2). The high jugular bulb in cases #1 and #3 had superior diverticula. The bony bottom of each PMDSS was extremely thin.

**Fig. 3:**
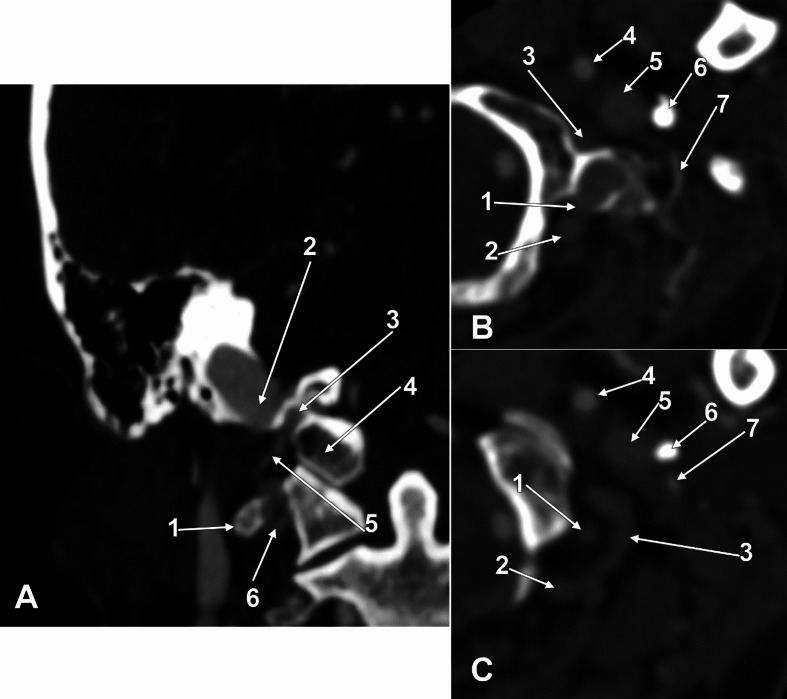
**A**. Coronal section through the paramastoid diverticulum of the right sigmoid sinus (PMDSS) in case #1. Anterior view. (1) transverse process of the atlas; (2) PMDSS; (3) paracondylar groove; (4) occipital condyle; (5) lateral condylar vein; (6) venous plexus of the vertebral artery. **B**, **C**. Successive axial sections through the PMDSS in case #3. Inferior views. (1) tip of the PMDSS; (2) posterior condylar vein; (3) lateral condylar vein; (4) internal carotid artery; (5) internal jugular vein; (6) styloid process; (7) occipital artery

In case #1, the lateral condylar vein crossed the PMDSS medially to join the plexus of the vertebral artery infero-medially to the PMDSS. The tip of that PMDSS reached 3.48 mm superior to the transverse process of the atlas. In case #2, the lateral condylar vein also coursed medially to the PMDSS through a deep paracondylar groove between the occipital condyle and the PMDSS (Fig. 3A).

In cases #1 and #2, the occipital artery coursed beneath the occipitomastoid fissure on the lateral side of the PMDSS.

In case #3, the tip of the PMDSS herniated through the bottom of the bony diverticulum to contact extracranially the posterior condylar vein on that side (Fig. 3B and C). The lateral condylar vein laterally surrounded the PMDSS and joined the posterior condylar vein suboccipitally. The occipital artery coursed laterally to both the PMDSS and lateral condylar vein.

## Discussion

An embryological basis for these findings may not be possible. The paramastoid location of the PMDSSs we found may point to a disturbance during the regression of the proatlas. However, no other anomalies of the craniocervical junction were found here. The PMDSS aetiology may better be related to the ossification process of the occipital bone and a focally issued tissue weakness.

The SS has a known diverticulum called the *sigmoid sinus plate recess* or simply the *sigmoid plate recess*. This diverticulum is formed by the inner surface of the mastoid part of the temporal bone– the SS plate or wall [7]. This recess can vary in size and shape across individuals and may have clinical relevance. Information on the recesses of the SS usually misses [1, 9]. In Bergman’s Encyclopedia of Human Anatomic Variations are listed two peculiar variants of the SS, a superior outpouching or diverticulum of the sigmoid sinus and, respectively, a blind end of the sinus that drained via a large mastoid foramen are listed [2]. Thus, it appears that the PMDSS is a novel finding. The dominance of the right SS is known [10], which may explain why 2 of our 3 cases had a right PMDSS.

The risk may be significant when not distinguishing the PMP from a PMDSS. This is because trauma or resection of a PMDSS, supposed to be just a PMP, could lead to an important lesion of the SS and bleeding. Such an outpouching of the SS could promote thrombus formation, potentially triggered by a neighbouring inflammatory or neoplastic process. In case #3, direct contact between the PMDSS and condylar veins was found. This possibility and the close relation of the PMDSS and the occipital and vertebral arteries recommend a preoperative check for the anatomical structures when a suboccipital approach is intended and designed.

The main limitation of this brief study is that only the PMDSSs were documented. New studies should evaluate the complete variational pattern of the SS.

## Data Availability

No datasets were generated or analysed during the current study.
